# Long-term performance of an atrial lead capable of accelerometer based detection of cardiac contractility in patients receiving cardiac resynchronisation therapy

**DOI:** 10.1371/journal.pone.0222269

**Published:** 2019-09-09

**Authors:** Thomas Senoner, Fabian Barbieri, Georg Semmler, Agne Adukauskaite, Andrea Rubatscher, Wilfried Schgör, Markus Stühlinger, Axel Bauer, Bernhard Erich Pfeifer, Lukas Fiedler, Franz Xaver Roithinger, Florian Hintringer, Alois Suessenbacher, Christian Georg Wollmann, Wolfgang Dichtl

**Affiliations:** 1 University Clinic of Internal Medicine III (Cardiology and Angiology), Medical University Innsbruck, Innsbruck, Austria; 2 Department of Internal Medicine III, University Clinic St. Pölten, St. Pölten, Austria; 3 Karl Landsteiner University, St. Pölten, Austria; 4 Landesinstitut für Integrierte Versorgung, Tirol Kliniken GmbH, Innsbruck, Austria; 5 Austrian Institute of Technology, Center for Health & Bioresources, Digital Health Information Systems, Eduar Wallnöfer Zentrum 1, Hall in Tirol, Austria; 6 Landesklinikum Wiener Neustadt, Department of Internal Medicine III, Wiener Neustadt, Austria; 7 St. Vinzenz Hospital, Department of Internal Medicine, Zams, Austria; University Medical Center Groningen, University of Groningen, NETHERLANDS

## Abstract

**Objectives:**

To evaluate the long-term performance of the SonRtip atrial lead.

**Background:**

To optimize atrioventricular and interventricular timing and thereby potentially improving cardiac resynchronization therapy (CRT) responder rates, a lead integrated technology and a cardioverter/defibrillator-based algorithm measuring peak endocardial acceleration have been introduced. Long-term performance of the atrial lead (SonRtip PS55D, Sorin/MicroPort CRM, Italy) embedded with such a sensor has not been reported so far.

**Methods:**

Between 2012 and 2018, 143 patients underwent implantation of the SonRtip atrial lead in four Austrian medical centers. Conventional bipolar atrial leads implanted during the same period in 526 patients receiving CRT were used as control cohort.

**Results:**

Among 669 patients included in the study, 10 (1.5%) showed increased atrial pacing thresholds and/or decreased atrial sensing amplitudes and/or sudden increase in atrial lead impedance (above 3000 Ω) after an uneventful early postoperative period. Seven (70%) of the malfunctioning leads were SonRtip leads (p <0.001). Lead replacement was needed in 4.2% of SonRtip leads (six out of 143) and in 0.38% of all other conventional atrial leads (two out of 526) (p <0.001). Because of unaltered atrial sensing properties, a wait and see strategy was chosen in two patients–one of them with a SonRtip lead. The implanted atrial lead in the latter person experienced a sudden increase in pacing threshold (4V/0.35ms).

**Conclusions:**

While short-term safety and stable technical performance of the SonRtip atrial lead could be confirmed, our study found an unexpectedly high malfunction rate over a longer follow-up period.

## Introduction

Responder rate of cardiac resynchronization therapy (CRT) is still disappointing at around 60 percent [1]. Optimized programming of atrioventricular (AV) and interventricular (VV) intervals is an option to improve CRT effectiveness. Optimization has classically been performed using echocardiography. However, this is time-consuming and rarely used in clinical routine. Therefore, alternative approaches for CRT optimization are needed. Device-based algorithms for optimization of CRT programming have been introduced in recent years, mostly using intracardiac electrogram (IEGM)-based techniques [2].

The only non-IEGM-based approach assesses peak endocardial acceleration generating a so-called SonR signal, which corresponds to the first heart sound (S1). The first component of the SonR signal is recorded during the isovolumetric contraction of the left ventricle. Its amplitude correlates with the contractile function (dP/dt_max_) [2]. The device optimizes VV and AV intervals on a weekly basis according to the highest average SonR signal, both at rest and during physical exercise.

Originally, this contractility sensor was part of a lead used for right ventricular pacing. This lead was tested in the CLEAR study showing a trend towards increased CRT response [3]. Thereafter, this sensor was embedded into a newly designed bipolar atrial screw in lead entitled SonRtip. This technology has recently been tested in a large prospective, randomized double-blind multicenter trial in patients with an established indication for a CRT-D device. In this RESPOND-CRT study, both short-term efficacy and safety could be proven. The rates of lead complications were 1.5% during the first 3 months after implantation (mainly lead dislocations) and 0.2% during the following 9 months [4].

Between 2012 and 2018, 143 SonRtip leads were implanted in patients receiving Sorin CRT-defibrillators at four Austrian centers. No lead-related adverse events occurred in the early postoperative period.

A previous study has evaluated the short-term (up to 1 year) safety and performance of the SonRtip atrial lead [5]. However, long-term data beyond 1 year have not been reported yet. Therefore, the aim of the current observational, multicenter trial was to assess the long-term performance of the SonRtip atrial lead.

## Methods

### Patient characteristics

Between 2012 and 2018, 669 patients with de novo implantation of CRT devices (permanent pacemakers and defibrillators) with 3 leads, or with an upgrade to CRT with implantation of atrial and transvenous LV leads were included in this observational, multicenter study.

Patients are followed on a regular basis either at the implanting institutions or by their referring cardiologists approximately every six months. An atrial lead malfunction was defined as an electrical problem concerning the atrial lead (increased atrial pacing thresholds and/or decreased atrial sensing amplitudes and/or sudden increase in atrial lead impedance [above 3000 Ω]) (e.g., lead fracture, insulation defects). Furthermore, atrial lead dislocations and other periprocedural complications were recorded (such as atrial perforation or infection).

All data were collected by the treating of physicians and fully anonymized before analysis. The ethics committee of the Medical University of Innsbruck waved the requirement for informed consent in a general statement concerning retrospective studies.

### SonRtip lead (Model PS55D)

The SonRtip is a bipolar, active fixation atrial pacing lead equipped with a cardiac contractility sensor designed for the use with Sorin/MicroPort defibrillators having a specific algorithm for measurements and analyses of cardiac contractility. The SonR sensor is contained in a hermetically sealed cylindrical titanium capsule placed near the tip of the lead. The sensor is a micro-accelerometer capable of measuring heart muscle acceleration. The electronic circuit transmits the signal to the SonRtip compatible defibrillator.

### Statistical analysis

Statistical analysis was performed using SSPS software (*V21*.*0*, *IBM SPSS Inc*., *Chicago*, *USA*), while graphs were designed using GraphPad PRISM, version 5 (GraphPad Software, Inc., La Jolla, CA, USA). Quantitative variables are expressed as means ± standard deviation (SD), categorical variables as absolute values and percentages.

Differences in all parametric data between two groups were tested using the independent t-test in case of normal distribution or Mann-Whitney U test for non-normally distributed and rank-scaled variables. To assess the distribution, the Kolmogorov-Smirnov test and histograms were used. Differences in categorical data were determined with Chi-Square or Fisher’s exact test (if n <5 per group).

Data were assessed prospectively during clinical routine and were retrospectively analyzed after they were retrieved from digital patient logs.

Survival curves were generated using the Kaplan Meier survival plots. All-cause mortality and survival free of lead malfunction was compared between the SonRtip atrial lead and the remaining atrial leads using the Log-Rank (Mantel-Cox) test. A *p-*value less than 0.05 was considered statistically significant.

## Results

### Technical performance of the SonRtip atrial lead

Between 2012 and 2018, 669 patients who were implanted with CRT devices (pacemakers: n = 110 and defibrillators: n = 559) with 3 leads or were upgraded with implantation of an atrial and transvenous LV lead were investigated in this multicenter observational study.

SonRtip and Beflex RF45D atrial leads from Sorin/Microport were used in 143 patients (21.38%) and five patients (0.75%), respectively. Medtronic CapSure Fix Novus 5076 and 4076 atrial leads were implanted in 110 patients (16.44%) and 52 patients (7.77%), respectively. St. Jude Medical/Abbott Tendril STS 2088TC and STS 1888TC atrial leads were implanted in 94 patients (14.05%) and two patients (0.3%), respectively. Flextend 4096 and Ingevity 7741 atrial leads from Boston Scientific were used in ten patients (1.49%) and 72 patients (10,76%), respectively. Biotronik Solia S53, Safio S53 and Tilda R53 leads were implanted in 104 patients (15.55%), five patients (0.75%) and one patient (0.15%), respectively. Atrial leads 4470, 4480 and 7736 from Guidant/Boston Scientific were used in 25 (3.74%), two (0.3%) and five patients (0.75%), respectively. SelectSure 3830, CapSure 4574 and 4592 atrial leads from Medtronic were used in ten (1.49%), five (0.75%) and 15 patients (2.24%), respectively. Finally, Abbott IsoFlex Optim 1944 and Tendril LPA1200M atrial leads were implanted in one patient (0.15%) and eight patients (1.20%), respectively.

### Overall atrial lead performance

Performance of SonRtip leads was compared with the performance of all other atrial leads. In the four Austrian centers who participated in the study, a total of 143 SonRtip atrial leads were implanted. After a median follow-up of 2.34 ± 1.91 years, 10 lead malfunctions (1.5%) were observed in 669 patients. Among them seven malfunctions occurred in SonRtip atrial leads (70%), while the remaining lead malfunctions occurred as follows: one lead malfunction in a Boston Ingevity 7741 atrial lead, one in a Biotronik Solia S53 atrial lead, and one in a CapSure SP Novus 4592 atrial lead.

**Table 1** shows the patients’ baseline characteristics between the two groups. Characteristics of patients with atrial lead malfunction are shown in **Table 2**.

**Table 1:** Characteristics of the studied population.

Baseline characteristics	*SonRtip* (n = 1*43*)	*Non-SonRtip* (n = 5*26*)	P-value
***Demographics***			
** Mean age (years)**	68.03 ± 9.4	70.07 ± 10.4	0.428
** Male sex, n (%)**	106 (74.1)	387 (73.6)	0.894
***Intraoperative measurements***			
** P-wave (mV)**	2.63 ± 1.9	2.87 ± 1.6	0.042
** Impedance (Ω)**	429.6 ± 95.3	557.76 ± 118.1	0.003
** Pacing threshold (V)**	0.84 ± 0.5	0.87 ± 0.5	0.001
***Device***			
** CRT-P**	0 (0)	110 (100)	< 0.001
** CRT-D**	143 (25.6)	416 (74.4)	< 0.001

Values are mean ± SD or n (%)

CRT-D: defibrillator; CRT-P: pacemaker; mV: millivolt; Non-SonRtip: Non-SonRtip atrial leads; Ω: Ohm SonRtip: SonRtip atrial leads; V: Volt

**Table 2:** Characteristics of patients with atrial lead malfunction.

gender / age at malfunction	lead type	days until lead malfunction	chest x-ray	atrial sensing (mV)	lead impedance (Ω)	pacing threshold (V)	consequences
**M 81**	SonRtip	830	Unremarkable	0.4	> 3000	0.25	Lead replacement
**M 80**	SonRtip	1439	No chest x-ray obtained at time of malfunction	0.4	> 3000	0.5	Lead replacement
**M 68**	SonRtip	1288	Unremarkable	0.6	> 3000	4.5	Lead replacement
**M 78**	SonRtip	718	No chest x-ray obtained at time of malfunction	0.7	> 3000	0.75	Lead replacement
**M 61**	SonRtip	840	No chest x-ray obtained at time of malfunction	2.2	> 3000	0.75	Lead replacement
**F 57**	SonRtip	904	Unremarkable	Not measurable	> 3000	Not measurable	Lead replacement
**M 76**	Ingevity 7741	1166	Unremarkable	1.4	523	5	Lead replacement
**F 49**	Solia S53	398	Unremarkable	1.5	> 3000	Not measurable	Patient underwent heart transplant
**M 77**	SonRtip	876	Unremarkable	2	466	4	Lead has not been replaced yet
**M 84**	CapSure SP Novus 4592	1181	Unremarkable	1.6	448	Not measurable	Lead replacement

mV: millivolt; Ω: Ohm; V: Volt

### Postoperative atrial lead performance

An early dislocation of an atrial lead occurred in three patients on the first postoperative day. One patient had a late micro dislocation and the lead had to be repositioned 287 days after the primary procedure. One patient had a right atrial perforation, and one patient had a pocket infection. None of the patients necessitated an atrial lead replacement. No atrial lead dislocations occurred in SonRtip atrial leads.

### Late atrial lead performance

The median time to atrial lead malfunction was 2.19 ± 1.11 years. Eight lead malfunctions displayed an increased lead impedance, five displayed decreased atrial sensing and four increased pacing thresholds. Four leads showed an unaltered pacing threshold, while the other two leads showed a complete exit block. Chest x-ray examination, performed in six out of nine patients at the time of lead malfunction, was unremarkable in all six patients. Atrial lead replacement was necessary in 8 out of 10 patients (six SonRtip atrial leads, one Boston Ingevity 7741, and one Medtronic CapSure 4592 atrial lead). A watch-and-wait approach was chosen in two patients with increased atrial pacing thresholds (around 4V at 0.4 ms) but still normal sensing properties and impedance (one SonRtip and one Biotronik Solia S53 lead, the latter in a patient who underwent heart transplantation within one year).

**Fig 1:**
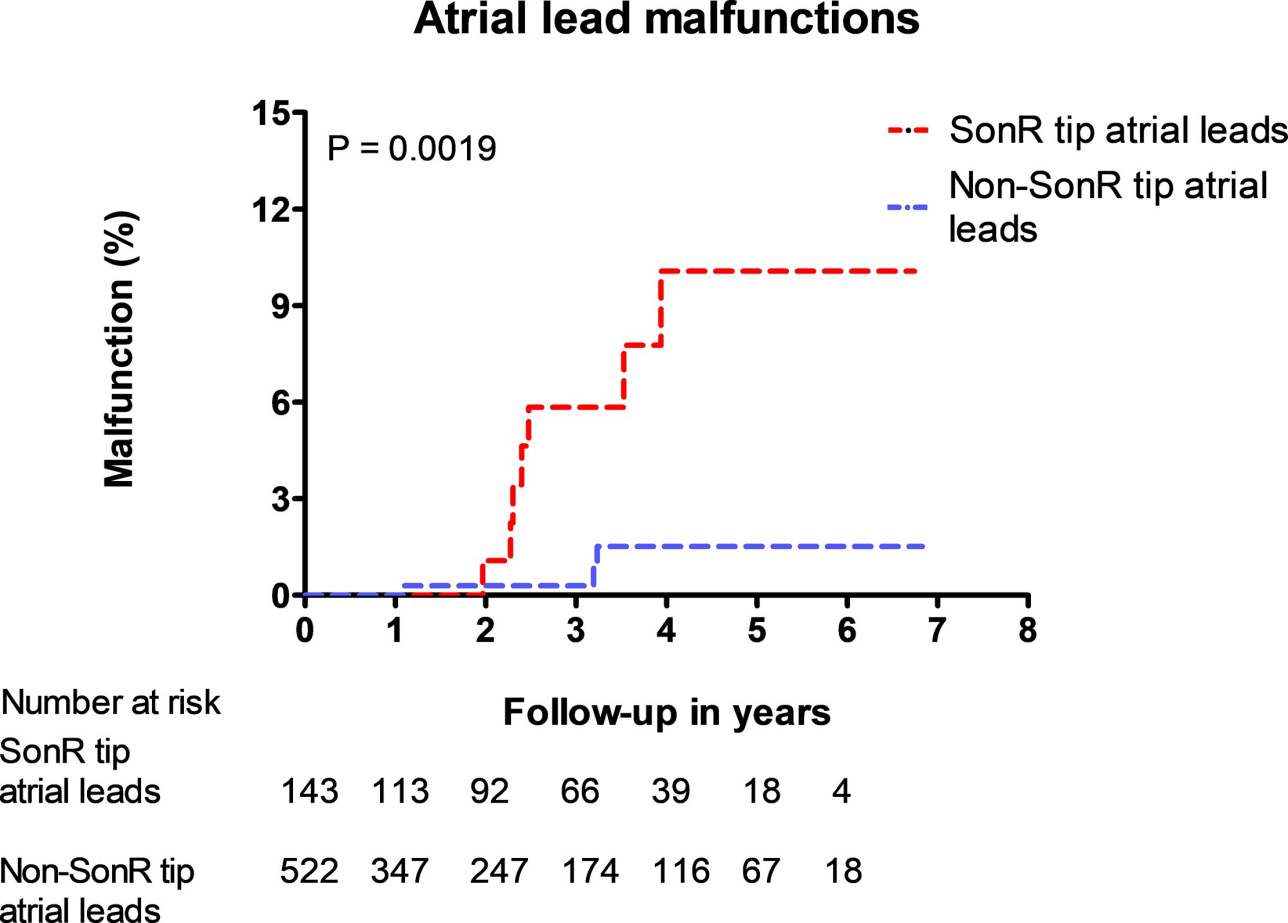
Kaplan Meier curve comparing the time of occurrence of malfunctions between SonR tip and non-SonR tip atrial leads.

Fig 1 displays the time course of the occurrence of malfunction comparing SonRtip with other atrial leads. More atrial lead malfunctions in SonRtip compared to other leads were observed, which was statistically significant (p = 0.0019). Among 143 implanted SonRtip leads, seven (4.9%) showed a malfunction after a median follow-up of 2.7 years. The incidence of lead malfunction remained constant between year 2 and 4.

**Fig 2:**
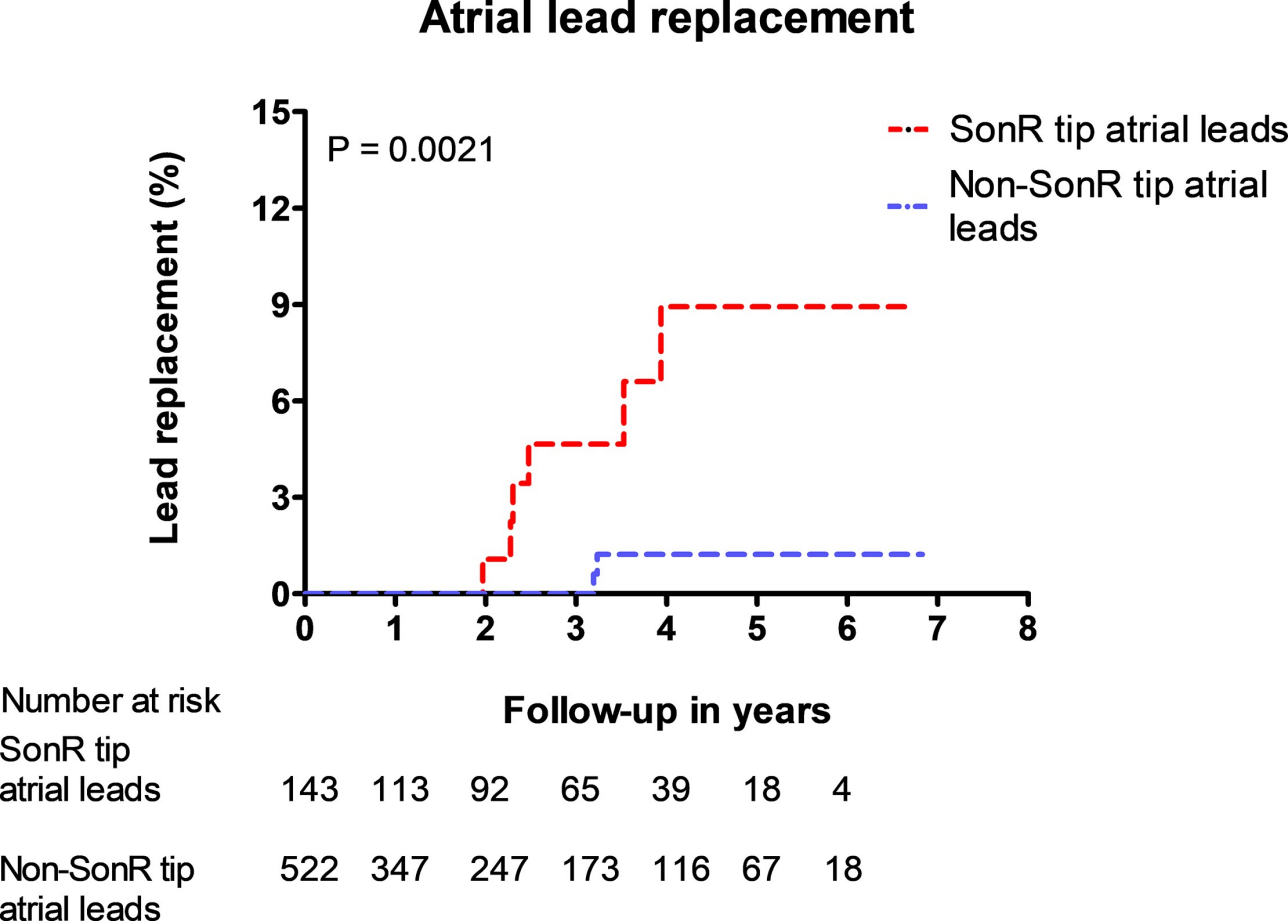
Kaplan Meier curve comparing the time of lead replacement between SonR tip and non-SonR tip atrial leads.

Fig 2 displays the time course of the need for lead replacement comparing SonRtip leads with other atrial leads. More atrial lead replacements in SonRtip compared to other atrial leads were observed, which reached statistical significance (p = 0.0021). Among 143 implanted SonRtip leads, six (4.20%) had to be replaced after a median follow-up of 2.7 years.

**Fig 3:**
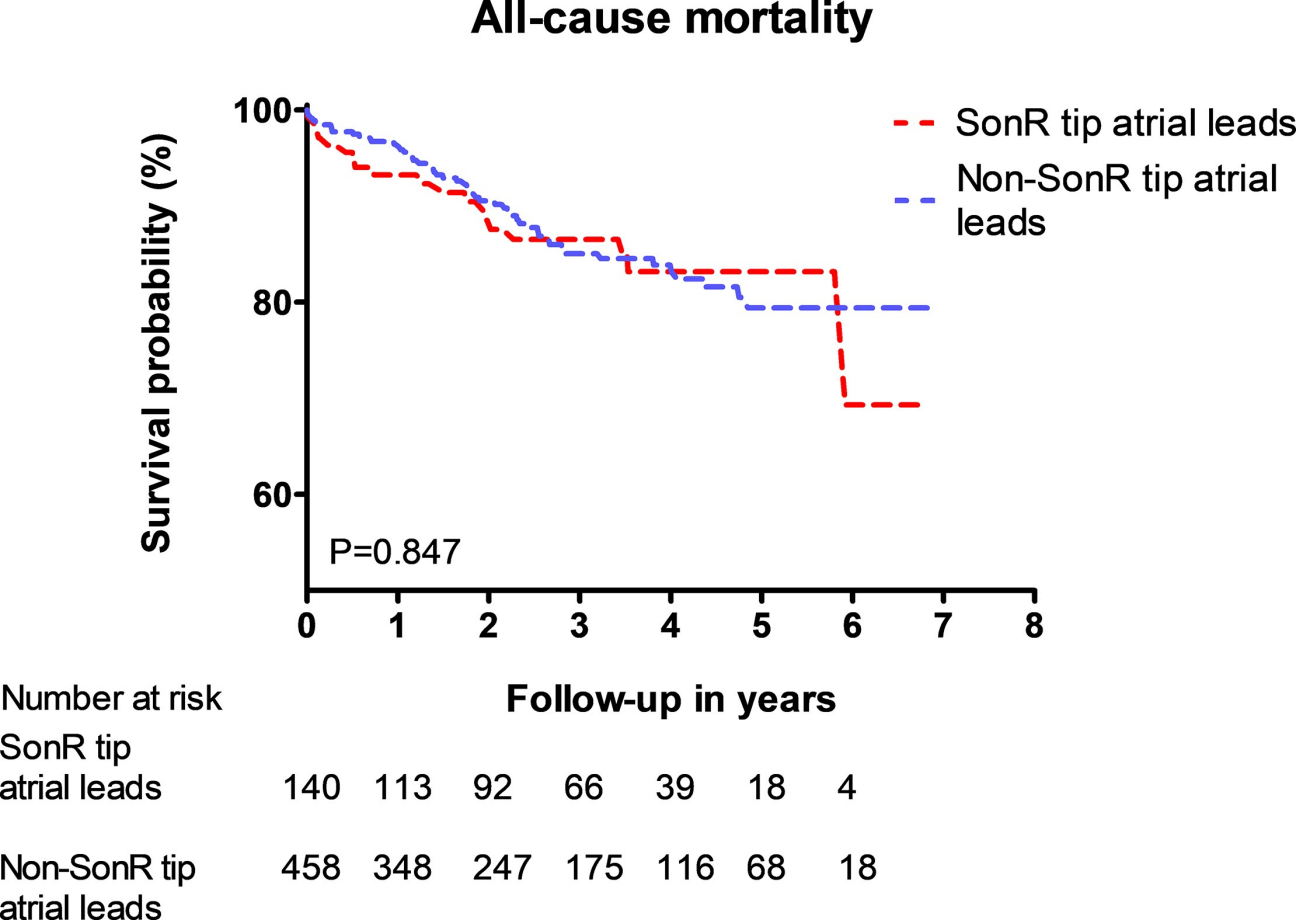
Kaplan Meier surival curve for all-cause mortality between CRT patients with SonR tip and non SonR tip atrial leads.

Fig 3 shows the Kaplan Meier survival curve for all-cause mortality. During the follow-up, 75 (11.21%) patients died: 19 with SonRtip atrial leads (13.28%) and 56 (10.65%) with non-SonRtip atrial leads (p = 0.847). Survival probability of patients with the SonRtip leads was unaffected compared to patients with non-SonRtip atrial leads.

## Discussion

Here we report an observational study of 669 consecutive patients who received new right atrial electrodes upon implantation of a cardiac resynchronization therapy device between 2012 and 2018 in four Austrian centers. These institutions introduced a newly designed atrial lead model called SonRtip which is supposed to improve CRT effects. After an uneventful postoperative period of two years, an unexpected high malfunction rate in long-term follow-up of this bipolar, active-fixation atrial electrode was noted.

Safety and long-term performance of newly introduced features of cardiac implantable electronic devices are key issues, since the risk of any complication following a CRT-D implantation has been reported to be almost 18% in a large Danish cohort study. Any lead replacement in CRT patients has been associated with a six month cumulative complication rate of almost 25% [6].

This lead has an embedded sensor at its tip measuring peak endocardial acceleration. Here we report that out of 143 patients receiving the SonR electrode, six suffered from a sudden increase in lead impedance (above 3000 Ω) accompanied by increased pacing thresholds and decreased sensing properties after an uneventful early postoperative period, whereas one lead showed increased pacing thresholds (4 V) while the impedance remained within the normal range (466 Ω). Based on our observational data, a sudden increase in impedance seems to be the main characteristic of malfunction in SonR tip atrial leads.

Recently ICD electrodes such as Sprint Fidelis (Medtronic Inc) or Riata (Abbott Inc.) caused significant harm due to increased rates of technical failures, in particular lead fractures [7]. A single-center study reported increased inappropriate noise sensing in conventional atrial leads from the Tendril family (Abbott Inc.) [8].

A sudden increase in impedance levels suggests a kind of lead fracture rather than an insulation defect. However, x-ray examinations (unfortunately not performed in all patients) did not reveal any visible abnormalities.

## Conclusion

In conclusion, we detected an unexpectedly high technical malfunction rate of the atrial SonRtip electrode in a small case series. Larger and thorough follow-up studies are needed to analyze the technical performance of this lead in clinical routine.
